# Women’s viewpoints on egg freezing in Austria: an online Q-methodology study

**DOI:** 10.1186/s12910-020-00571-6

**Published:** 2021-01-06

**Authors:** Johanna Kostenzer, Antoinette de Bont, Job van Exel

**Affiliations:** 1 Erasmus School of Health Policy and Management, Erasmus University Rotterdam, P.O. Box 1738, 3000 DR Rotterdam, The Netherlands; 2 Erasmus School of Economics, Erasmus University Rotterdam, P.O. Box 1738, 3000 DR Rotterdam, The Netherlands

**Keywords:** Egg freezing, Social egg freezing, Oocyte cryopreservation, Viewpoints, Ethics, Q-methodology, Austria

## Abstract

**Background:**

Egg freezing has emerged as a technology of assisted reproductive medicine that allows women to plan for the anticipated loss of fertility and hence to preserve the option to conceive with their own eggs. The technology is surrounded by value-conflicts and is subject to ongoing discussions. This study aims at contributing to the empirical-ethical debate by exploring women’s viewpoints on egg freezing in Austria, where egg freezing for social reasons is currently not allowed.

**Methods:**

Q-methodology was used to identify prevailing viewpoints on egg freezing. 46 female participants ranked a set of 40 statements onto a 9-column forced choice ranking grid according to the level of agreement. Participants were asked to explain their ranking in a follow-up survey. By-person factor analysis was used to identify distinct viewpoints which were interpreted using both the quantitative and the qualitative data.

**Results:**

Three distinct viewpoints were identified: *(1) “women should decide for themselves”*, *(2) “we should accept nature but change policy”, and (3) “we need an informed societal debate”*. These viewpoints provide insights into how biomedical innovations such as egg freezing are perceived by women in Austria and illustrate the normative tensions regarding such innovations.

**Conclusions:**

Acknowledging the different prioritizations of values regarding assisted reproductive technologies is important to better understand the underlying normative tensions in a country where egg freezing for social reasons is currently not allowed. The study adds new empirical insights to the ongoing debate by outlining and discussing viewpoints of those directly affected: women. Following up on the lay persons perspective is particularly important in the context of future biomedical innovations that may challenge established norms and create new tensions. It therefore also adds to the societal debate and supports evidence-informed policy making in that regard.

## Background

Preserving the option to conceive through egg freezing (or oocyte cryopreservation) is a technology in the field of assisted reproductive medicine which is increasingly gaining importance. In light of a diverse set of socio-cultural changes [1, 2], egg freezing has become a means to plan ahead for the anticipated loss of female fertility in a society where women have fewer children at a later age [3]. Following the publication of guidelines by international medical societies, egg freezing for social reasons has gained traction internationally [4, 5]. Ever since, several (multi-national) companies have started to include social egg freezing in employee’s benefit packages (mostly as part of a larger benefit package that also covers costs for adoption, IVF etc.) [6]. The controversy around this approach was not only followed by media attention but also created scientific interest. The use of the technology, therefore, continues to be subject to ongoing discussions. Whereas egg freezing for medical reasons, as in the context of cancer treatment or genetic predispositions impacting fertility, is considered a legitimate and established option, egg freezing for non-medical and often called social reasons causes more conflicting reactions which can be linked to different value judgements [7, 8].

Ethical issues that arise in assisted reproductive medicine and fertility preservation have been addressed by various scholars in the past from both normative [9–11] and empirical perspectives [12, 13]. Underlying norms and value perceptions shape how biomedical innovations such as assisted reproductive technologies are perceived, leading to a diverse set of objections against and arguments for egg freezing that are not without controversy [5, 14–17]. On the one hand, arguments related to unnaturalness, biomedicalization of a societal problem, and negative impacts on society are complemented by concerns related to the actual performance of the procedure [14, 18]. On the other hand, arguments in favour of egg freezing focus on the potential positive effects in terms of reproductive autonomy, gender equality, psychological benefits and the reduced need for third-party involvement in assisted reproduction [14, 19].

The topic of egg freezing as a fertility preservation method has furthermore been addressed from various social science disciplines. Users’ profiles as well as their motivations to engage in egg freezing have been explored in various contexts [20–24]. As has been shown in previous research, women who engage in egg freezing are mostly single and in a period of their life when their fertility has already started to decline [24, 25]. Their reasons to engage in egg freezing are potentially manifold, and are shaped by a complex set of physical, economic, structural, and relational factors [20]. It was found that rather than being attributed to one dominating reason, women’s decisions to freeze eggs result from the absence of a set of conditions considered essential for pursuing parenthood [20]. Moreover, attitudes towards assisted reproductive technologies including fertility preservation have been investigated in various countries [8, 26–31]. The findings show that knowledge of the technology and also acceptance of its use have been increasing in the past [26, 29]. While the existing studies provide valuable insights into women’s attitudes towards certain aspects of egg freezing for both medical and social reasons, our research explores the prevailing viewpoints on egg freezing in a systematic manner. This study focuses on a setting where egg freezing for social reasons is currently not allowed and where the use of the technology is therefore particularly controversial.

Engaging in the ethical reflection of egg freezing and having knowledge about who “the freezers” and what their motivations are is important to understand the implications involved, and to provide good quality of care by developing ethically sound policies in this regard. What is so far unknown, however, is how the normative tensions related to egg freezing are perceived by those who (potentially) engage in egg freezing: women. Therefore, rather than by adding to the normative analysis from an expert view, this paper adds a new perspective by empirically investigating lay persons viewpoints on egg freezing. With that, this paper aims to reflect on the normativity that surrounds egg freezing as perceived by women and gives context specific insights into their realities.

The use of empirical methods has been gaining importance in the field of medical ethics [32] in context of the “empirical turn” and the increasing use of social scientific methods in bioethics [33]. Particularly the identification and analysis of different stakeholders’ perceptions has increased in significance within the now called field of “empirical bioethics” [34]. Empirical bioethics, in this context, is considered an interdisciplinary integration of empirical social science and ethical analysis. By conducting this study on the topic of egg freezing, we engage in empirical-ethical research and will link certain aspects of our findings to the ethical debate in our discussion. We use Q-methodology for this purpose and focus our study on women’s viewpoints in Austria. As one of a few countries in Europe—next to e.g. France, Hungary, Lithuania, Malta, Norway, Serbia and Slovenia—egg freezing for non-medical reasons is not allowed in Austria [35]. The Q-methodology approach has been used before to explore ethically sensitive topics, as for example end-of-life decisions in healthcare [36, 37] or organ donation [38] and is also considered useful for the topic of egg freezing.

In our study, we use the terms “medical” and “non-medical” or “social” egg freezing. We are aware that different conceptualisations of egg freezing [e.g. as (non)medical or social, preventive measure, elective treatment, or as an act of self-donation] create different implications and are not value-free. However, as the terms chosen are commonly used in the Austrian public discourse and by the lay public^1^—which is at the centre of this study—we refer to them in the context of our research.

### The Austrian context

Assisted reproductive medicine is regulated under the Austrian Law on Reproductive Medicine [39] and under the IVF Fonds Act [40]. Despite a thorough revision of the Law on Reproductive Medicine in 2015, which represented a shift to a more liberal and permissive regulatory approach, egg freezing for non-medical reasons is still not allowed in Austria, both in terms of general access and coverage [41]. Egg freezing for medical reasons, however, is allowed for certain indications irrespective of age, yet not covered by the social health insurance [35].

The amendment process of the law was accompanied by controversial debates on many topics yet also on egg freezing, involving experts from various disciplines. The Austrian Association for Gynecology and Obstetrics for example promoted egg freezing in its commentary as “preventive freezing of egg cells and sperm freezing” [42]. Efforts by the Association and other parties advocating the legalization of social egg freezing, however, did not receive much attention by the regulators and it remains unclear why egg freezing for other reasons than apparent medical ones is still not allowed and for this reason also not available [43, 44]. Relating specifically to social egg freezing, the Austrian Bioethics Commission, which acts as an advisory board to the Austrian Chancellery, supported the restriction of social egg freezing at the time [45]. The Commission published a statement as part of a position paper on the amendment in which it refers to the risk of encouraging young women to postpone their desire to have a child which could be achieved earlier. In a TV discussion round a few years later,^2^ however, Dr. Christiane Druml, head of the Bioethics Commission, expressed a more supportive opinion and favoured the legalization of social egg freezing also in Austria. The restrictive approach to egg freezing in Austria can be partly explained by the general eligibility criteria for assisted reproduction. So far, fertility care may only be accessed by couples—if all other reasonable means of achieving pregnancy were not successful [46]. 70% of the IVF costs of up to 4 cycles of treatment are covered if the respective couples qualify according to the Austrian IVF Fonds Act. However, coverage only applies if the woman is not more than 40 years old and the man not more than 50 years old [40]. Single women are hence excluded, both from access to ART and coverage, pointing towards a prioritization of the regulator of a more traditional concept of family and upbringing. Medical egg freezing is generally not covered by the Austrian social health insurance [40]. However, part of the costs, such as medication, might be covered on a case-by-case basis, depending on the decision by the insurer. As social egg freezing is not allowed, it is also not funded.

At the same time, however, this does not mean that (single) women in Austria are not demanding social egg freezing, yet those interested need to seek the service abroad. Official data or even estimates about this form of cross border reproductive care are not being collected and are unavailable. Informal conversations with experts let assume, however, that a few women engage in egg freezing abroad. Several Austrian fertility clinics do also operate in neighbouring countries as in Liechtenstein or the Czech Republic, where this treatment is not prohibited, and may refer interested women to seek the service there. It was found that those who engage in reproductive travel do so to evade restrictive legislation [47]. Reproductive travel for the purpose of social egg freezing is not forbidden in Austria, however, it still does not come without challenges. Particularly cross border fertility preservation also creates additional burdens, not only regarding different legal regulations making the future usage of the frozen eggs more complex, but also in terms of the higher emotional burden and costs. The discrepancy between policy-makers restricting access to the technology on the one side, and some women demanding the service (and accessing it in other countries) on the other side, however, gives food for thought and highlights the need for further research and debate.

## Methods

Viewpoints on egg freezing depend on a diversity of factors, including normative perceptions and underlying value patterns regarding the life course, as outlined before. To explore this complexity further, we used Q-methodology [48], a mixed methods approach. We conducted the study online and collected data from 46 female respondents. These respondents shared their subjective viewpoints on egg freezing by ranking a set of 40 predefined statements and explaining their ranking of the statements. By-person factor analysis of the ranking data was used to identify shared viewpoints, which were further interpreted using the qualitative data.

### The statement set

The 40 statements used in this study were extracted from a variety of sources covering issues related to egg freezing (i.e. reproductive autonomy, equality, distributive justice, resource allocation). To cover the whole spectrum of the ongoing scientific and non-scientific debate on egg freezing, scientific literature, newspaper articles, policy documents, medical guidelines, interviews and informal talks as well as (social) media reports, blog posts, podcasts and discussions in online fora were consulted, and a small-scale expert survey was administered during a conference. A total of 91 statements was collected, which the first author structured into categories representing the most relevant aspects that shape opinions on egg freezing identified in the literature [5, 17, 49]. These included notions of age and timing, benefit and harm, biological boundaries, coverage, ethics and morality, justice, biomedicalization, ownership, reproductive autonomy, and work-life-balance. All authors then contributed to the development of the preliminary statement set and exchanged feedback in multiple discussion and revision rounds, during which redundant, ambiguous and unclear statements were combined, deleted or rephrased, leaving 43 statements. The preliminary statement set was presented and commented upon by experts at the workshop “Young Medical Ethics” at the Academy for Ethics in Medicine at University of Göttingen in January 2019 and at the expert symposium “Comparative and transnational perspectives on technologies of fertility preservation and extension” at De Montfort University in Leicester in June 2019. The English set was piloted (n = 8) in early 2019 resulting in small revisions of the statement set (e.g. regarding the wording of some statements) and a reduction to 40 statements. The statements as well as instructions were later translated into German by use of professional translation services, which was checked by the first author (the statement set was additionally used for a study conducted in the Netherlands in parallel to this one [50]). The German set was then again tested (n = 4) in July and August 2019. No further changes were required. The final set of 40 statements is included in Table 3.

### Data collection

Adult women (above the age of 18) currently living in or originating from Austria were the general target population for this study. By aiming at exploring women’s viewpoints on egg freezing—a reproductive technology that is targeted towards women—we did not set upper age limits or exclusively focus on those who might consider using the technology. Instead, we took a broader perspective to explore women’s viewpoints of all ages, and to identify most diverse opinions on the topic. For a Q-methodology study, a group of 40–60 purposively selected respondents is generally considered sufficient for identifying the diversity of views that exist on a topic [48]. In order to reach a large variety of respondents, covering the lay and more informed views on the topic, and hence increasing the odds of including women with different viewpoints on egg freezing, they were approached via multiple ways: targeted outreach to women who were known to have strong opinions on egg freezing, mouth-to-mouth recruitment, use of social media (e.g., announcements via Facebook, Twitter, LinkedIn and WhatsApp groups), use of professional and personal networks and via other interest groups.

Data collection occurred online using an updated version of FlashQ.^3^ In the invitation message that was sent to the participants, background information concerning the study and a link to the online tool was provided. After following the link, respondents reached a website with detailed instructions and information about anonymity and data use. By clicking on a “next” button, participants confirmed to have read and understood the information provided and to take part in the study. They were informed about the opportunity to stop participation at any time. In this case, data was not saved and hence not included in the study.

**Fig. 1:**
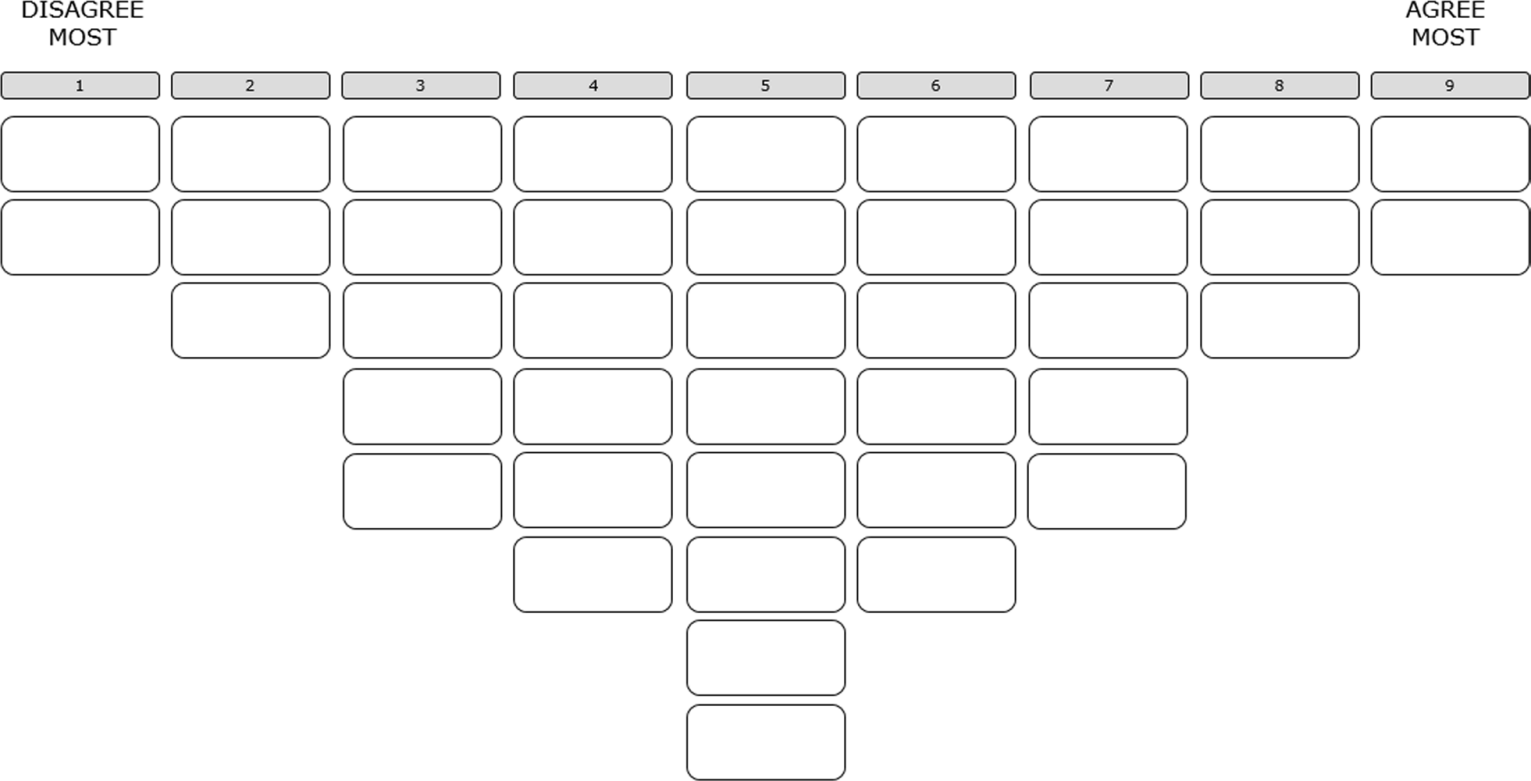
9-scale Q sorting grid

During the online data collection, respondents were presented with the set of statements on egg freezing. First, they were asked to read all the statements and to divide them into three piles (agree, neutral or do not know, disagree). Next, they were asked to place them on a forced-choice sorting grid ranging from 1 “disagree most” to 9 “agree most” (see Fig. 1), starting with the statements in the “agree” pile, followed by those in the “disagree” and “neutral or do not know” piles. Finally, after potentially making last changes to their ranking of the statements, the participants were asked to explain their ranking, focussing on written comments to the statements placed in the two outer columns of the grid. Here, participants could outline the specific reasons for their choice. Their explanations were used later to support the interpretation of the statement ranking. In addition, the participants received questions regarding demographic details (e.g. name, educational level, relationship status, number of children, experience with egg freezing and potential willingness to engage in egg freezing) and could give general comments on the study. At the end of the data collection process, participants submitted their data and were forwarded to the contact page of the first author of this study in case of follow-up questions.

### Analysis

In order to identify prevalent viewpoints on egg freezing among women in Austria, the 46 rankings of the statements were analysed using PQMethod [51]. A correlation matrix of all pairwise correlations between the rankings of the statements by respondents was computed, and factor analysis (i.e., centroid factor analysis followed by varimax rotation) was used to identify participants with mutually high correlations (and low correlations with other participants). The analysis showed that solutions of up to four factors were supported by the data. We examined the statistical characteristics and interpreted the factors in solutions with three and four factors, also considering the qualitative materials collected from the respondents. Afterwards, the solution with three factors was selected because it was comprehensive and consisted of statistically strong (based on contribution to explained variance and number of associated respondents) and clearly interpretable factors [48]. Finally, a factor array was computed for each of the three factors. This means that, for each factor, weighted average rankings were computed for all 40 statements, based on the rankings of the statements by respondents associated with that factor and their correlation with the factor. The two statements with the highest average ranking were assigned a factor score of + 4 for that factor, the next three a factor score of + 3, and so on (according to the number of spaces in each column in the sorting grid; see Fig. 1). The factor scores thus represent in which column of the sorting grid a hypothetical respondent with exactly the view portrayed by the factor would have placed the statements. The holistic interpretation and description of the factors as distinct viewpoints on egg freezing was based on the resulting factor arrays and the qualitative data. Of particular interest were the characterizing statements (which are those with a factor score of − 4, − 3, + 3 or + 4 in the factor array) and the distinguishing statements (which are those with a significantly different factor score as compared to the other factors).^4^ Finally, quotes from the written comments given by the respondents associated with a factor were used to further interpret that factor and illustrate the viewpoints in more detail.

## Results

A total of 48 respondents completed the online survey. Two respondents did not meet the selection criteria—one identifying as male and one without any affiliation to the Austrian context- leading to a total of 46 respondents included in the analysis. The average age of the respondents is 36 years, ranging from 25 to 62 years, almost all employed and some students. Table 1 shows the demographic characteristics of respondents.

**Table 1:** Q study sample (n = 46 females)

Characteristics	N	%
*Age*		
< 26	3	7
26–30	10	22
31–35	13	28
36–40	11	24
41–45	3	7
46–50	2	4
> 50	4	9
*Highest educational level*		
High school, vocational training	8	17
Higher education (bachelor, master, Ph.D.)	37	80
NA	1	2
*Relationship status*		
Single	9	20
In a relationship	35	76
NA	2	4
*Children*		
Yes	18	39
No	28	61
*Egg freezing experience*		
No	46	100
*Would consider egg freezing*		
Yes	21	46
No	25	54

Three distinct viewpoints on egg freezing were identified with this research. Together these factors explained 49% of the variance in the rankings of the statements by respondents (i.e., 19%, 16% and 14%, respectively) and 42 women associated statistically significantly (*p* < 0.05) with one of the three factors (i.e., 19, 12 and 11, respectively). The correlations between factors was negligible for factors 1 and 3 with factor 2 (i.e., − 0.04 and 0.02, respectively) and considerable between factors 1 and 3 (i.e., 0.64), but sufficiently distinct and considering their interpretations interesting enough to retain and discuss as separate factors. The factor loadings of respondents on the three factors are presented in Table 2. The factor arrays, showing how a typical representative of each view would have ranked the statements, are presented in Table 3.

**Table 2:** Factor loadings (n = 46)

Resp	Factor 1	Factor 2	Factor 3
1	0.6971*	0.0601	0.4431
2	0.7013*	0.2419	0.1354
3	0.0299	0.7112*	0.0138
4	0.3907*	0.0273	− 0.3537
5	0.5011	0.2116	0.5342
6	0.3316*	0.0767	0.2914
7	− 0.0752	0.7083*	0.2556
8	0.5594*	− 0.2668	0.2481
9	0.3520	− 0.3042	0.6433*
10	0.4917	− 0.4544	0.3993
11	0.5627*	0.0317	0.0663
12	0.1683	0.6637*	− 0.3021
13	0.5306*	0.4815	0.2099
14	0.7003*	− 0.1947	0.0927
15	0.3218	0.2836	0.4853*
16	0.5935*	− 0.0502	0.2414
17	0.2919	0.4422*	0.2898
18	0.3842	− 0.0697	0.6337*
19	0.7228*	0.0089	0.2492
20	0.3571	0.2889	0.4839*
21	− 0.0373	0.7244*	− 0.0861
22	− 0.2085	0.7380*	0.0985
23	− 0.1227	0.6420*	0.2572
24	0.6111*	− 0.4183	0.2407
25	0.2384	− 0.0913	0.6218*
26	0.4800*	0.0822	0.2519
27	0.1962	0.0991	0.6547*
28	0.3200	0.2443	0.6975*
29	− 0.2377	0.7890*	0.0667
30	0.5742*	0.1426	0.1434
31	0.3884	0.4119	0.4319
32	0.2743	− 0.4041	0.6453*
33	0.5002*	− 0.2188	0.4287
34	0.3534	0.0107	0.5806*
35	0.5382*	− 0.0210	0.3255
36	0.1213	0.7044*	− 0.2813
37	0.1233	0.2815	0.4774*
38	0.6478*	− 0.1510	0.3135
39	0.6065*	− 0.1761	0.3400
40	− 0.2467	0.5932*	0.4951
41	0.7781*	0.0195	− 0.0306
42	0.3225	0.6677*	− 0.0581
43	0.0275	0.6893*	− 0.1525
44	0.0632	− 0.1805	0.3954*
45	0.2918	0.1850	0.0554
46	0.6329*	− 0.0817	0.2522

Statistically significant (*p* < 0.05), unique associations are marked with *

**Table 3:** Factor scores per statement

#	Statement	Factor		
		1	2	3
1	Egg freezing for social reasons stimulates women to postpone childbearing	− 1*	1*	0*
2	There should be strict age limits for assisted reproduction	2	2	1*
3	When women want to have children, they should do so at a younger age	− 3	2*	− 2
4	Women wait too long with starting a family	− 2*	0	− 1
5	The potential benefits are worth the burden of the treatment (hormone therapy and egg cell retrieval)	0	− 1*	1
6	Although the chance of success is uncertain, it is important that the option is available	2	− 1*	3
7	Egg freezing for social reasons creates false hopes about the ability to have children at a later age	− 2*	4*	− 1*
8	Freezing eggs is preferable over freezing embryos	2	0*	2
9	Women are insufficiently aware of their fertility lifespan	1*	0*	3*
10	Egg freezing for social reasons is a business of hope	− 1*	0	1
11	It is unnatural to preserve fertility beyond the fertile age	1*	4*	0*
12	Only because the option of freezing eggs is available does not mean it should be done	0	3*	− 1
13	Egg freezing for medical reasons should be covered by the health insurance	3	1*	2
14	Egg freezing for social reasons should be covered by the health insurance	− 1*	− 3*	− 1*
15	Egg freezing for social reasons should be paid for by the user	1	0	0
16	Women who want to freeze their eggs for social reasons should do so abroad	− 4	− 2*	− 3
17	The extension of fertility improves gender equality	0	− 4*	0
18	Egg freezing for social reasons promotes equal opportunities for women and men	0	− 2*	1
19	There is insufficient attention for the ethical aspects of egg freezing in general	0	1*	0
20	Egg freezing for social reasons requires a societal debate	0	0	2
21	I find egg freezing for medical reasons more acceptable than for social reasons	2	2	0*
22	Not all social reasons for egg freezing are equally good reasons	1	1	0*
23	Egg freezing is against my convictions	− 4	0*	− 4
24	Starting a family is an ultimate wish of human beings	− 1*	− 1*	− 2*
25	Egg freezing for social reasons should remain prohibited by law	− 3*	1*	− 3*
26	People have a right to have a genetic child	2*	− 1*	− 4*
27	People have a right to have a child	4*	− 2	− 3
28	Egg freezing should be available to all women	3	− 3*	3
29	Women should be able to freeze their eggs for any reasons	0	− 2*	2
30	Age-related fertility loss is a medical problem	− 2	− 2	1*
31	Women should make unused eggs available for donation	− 2	− 1	− 2
32	Unused eggs should remain in possession of the woman	1	0	2*
33	Women should be able to make their own choices regarding fertility preservation	4	− 1*	4
34	Egg freezing for social reasons allows women to organize their lives without pressure from the ‘‘biological clock.’’	1	− 4*	1
35	If infertile women want to have children, they should opt for adoption	− 1	1*	− 1
36	If women take the risk of waiting too long they should accept the possible consequence of childlessness	− 2*	3*	− 1*
37	It is difficult to invest in career and family at the same time	3	2	4*
38	Women should think of their career first and parenthood next	− 3	− 3	− 2
39	Political measures are needed to facilitate parenthood at a younger age	0*	3*	− 2*
40	Employers should facilitate parenthood at a younger age	− 1	2*	0

Statements which are characterizing for a factor are the ones scoring − 4, − 3, + 3, and + 4, distinguishing statements are marked with *

### Viewpoint 1: “Women should decide for themselves”

The emphasis on making own choices without restrictions is central to this viewpoint. Respondents strongly agree that women should be able to make autonomous choices regarding fertility preservation (#33, + 4) even though success rates might be low (#6, + 2). *“Women should be able to choose IF and WHEN they want to have children”* (Resp 8). Statements indicating that women should have children earlier (#3, − 3), that they wait too long with childbearing (#4, − 2), or that they should donate unused eggs, are therefore opposed (#31, − 2). Women sharing this viewpoint further think that the option to use egg freezing should be available to all women (#28, + 3). This is also in line with the strong agreement with the right to have a (biological) child (#27, + 4; #26, + 2). *“Sometimes the desire for a child and the life situation do not match, so this desire can be fulfilled at a more appropriate time”* (Resp 41). In this viewpoint, women who want to freeze their eggs for social reasons should be able to do so in Austria (#16, − 4). *“This treatment should be equally available in every country”* (Resp 35).

Women sharing this viewpoint perceive combining career and family as difficult (#37, + 3). However, they hold a rather indifferent position towards the need for political measures or measures by the employer to facilitate parenthood at a younger age (#39, 0; #40, − 1), which is again in line with the strong focus of this factor on autonomous choices and non-interference. *“Starting a family is a private thing. Every woman should be able to decide—on her own or together with her partner—when she is ready for becoming a mother. Involvement by the employer in these decisions is not acceptable as it would mean that the woman is not just an employee but also more or less property of the company, or at least treated as such”* (Resp 6).

Egg freezing is in general not in conflict with the respondents’ convictions (#23, − 4). However, this viewpoint shows a slight preference for medical over social freezing (#21, + 2), which is also reflected in stronger support for coverage of medical egg freezing than for social egg freezing (#13, + 3; #14, − 1).

### Viewpoint 2: “We should accept nature but change policy”

Contrary to the first viewpoint identified with this research, viewpoint two shows a more critical stance towards egg freezing. Respondents sharing this viewpoint perceive prolonging fertility beyond the fertile life age as unnatural (#11, + 4), and they think that women who take the risk of waiting too long should accept the possible consequence of childlessness (#36, + 3). *“One should abide by the laws of nature. If it is no longer possible to have children when you’re older, then this is completely natural and probably there is a solid, biological reason for that”* (Resp 36).

This viewpoint furthermore reflects a negative attitude towards possible societal improvements resulting from egg freezing, for instance regarding gender equality (#17, − 4; #18, − 2). These respondents do not think that egg freezing allows women to organize their lives without pressure from the “biological clock” (#34, − 4). Insurance coverage of social egg freezing is therefore also opposed (#14, − 3).

In viewpoint two, it is furthermore not believed that there is a right to have a (biological) child (#26, − 1, #27, − 2) and egg freezing should neither be available to all women (#28, − 3) nor for all reasons (#29, − 2). Strict age restrictions for assisted reproduction are considered necessary (#2, + 2). *“Freezing eggs should—if at all—only be allowed in exceptional cases”* (Resp 36). Egg freezing for social reasons is moreover perceived to create false hopes about the ability to have children at a later age (#7, + 4). *“It creates the impression that you can take time with having children. The fact that the natural, biological conditions are decreasing is being neglected”* (Resp 22). Respondents hence do not think that just because the option to freeze eggs is available it should also be done (#12, + 3).

Similar to viewpoint one, combining career and family is perceived to be difficult (#37, + 2). *“Women at older age face the same challenges regarding motherhood as at younger age. Because of the increasing age it is probably even more difficult to re-enter working life after pregnancy”* (Resp 3). Yet, contrary to the first viewpoint, women agree with the need for political and employment measures to facilitate parenthood at a younger age (#39, + 3; #40, + 2). *“In order for women to have children at younger age, it is necessary that education and working conditions change, especially also for the fathers. Raising kids is the responsibility of both parents. But if it remains more attractive that fathers work more than mothers, women will stay disadvantaged”* (Resp 29).

### Viewpoint 3: “We need an informed societal debate”

Viewpoint three is characterized by a permissive attitude towards egg freezing for any reasons (#6, + 3; #28, + 3; #29, + 2), and does*—*as the only viewpoint*—*not prioritize medical over social reasons (#21, 0). Egg freezing is not considered to conflict with the respondents’ convictions (#23, − 4), and therefore they also do not believe that shifting the issue to other countries by not allowing egg freezing in Austria is a sustainable solution (#16, − 3). *“This simply outsources the problem and makes the treatment more expensive”* (Resp 34). Just like viewpoint one, this viewpoint shows a strong emphasis for making own choices regarding fertility preservation (#33, + 4). *“Freezing eggs is another step towards more self-determination of women”* (Resp 9). Yet, unlike viewpoint one, it reflects strong disagreement with the right to have a (biological) child (#26, − 4; #27, − 3) and having a family is not believed to be an ultimate wish of human beings (#24, − 2).

Compared to the other viewpoints, these women are slightly more positive that allowing social egg freezing will promote equal opportunities for women and men (#18, + 1). Yet, as the only one, viewpoint three stresses the need for a societal debate about social egg freezing (#20, + 2). *“I think it is important and right to make use of the medical possibilities. At the same time, however, ethical considerations should of course be thought of”* (Resp 34). The importance of reflection is in line with the respondents’ perception that women are insufficiently aware of their fertility lifespan (#9, + 3). *“Medical education and health literacy are extremely bad in Austria. This has also been proven by many studies”* (Resp 20). The need for more education is stressed in this context: “*I think there is too little information available and as a woman you are simply not being educated well enough about this topic*” (Resp 32); “*The level of information is currently very low. What is needed is a societal debate based on facts*” (Resp 34).

Also in viewpoint three, the difficulty of combining career and family is strongly emphasized (#37, + 4). *“Since most states and their economies do not take responsibility for the reproduction of their societies, women mostly face a double burden. This known problem of combining career and family is important because it shows that women do not have the same opportunities on the labour market as men due to this double burden, because even if men have a family and a career, this is rarely seen as a similar challenge”* (Resp 5). At the same time, participants do not think that political measures are needed to facilitate parenthood at a younger age (#39, − 2) and are neutral regarding the employers’ responsibility in doing so (#40, 0). *“Political intervention would again restrict women’s self-determination”* (Resp 9).

## Discussion

Egg freezing has emerged as an assisted reproductive technology for women to preserve the option to conceive. Be it for medical or non-medical reasons, egg freezing has led to heated debates among experts and the broader public alike. Scholars have in this context extensively explored ethical issues [5, 14, 17–19] and women’s motivations to engage in egg freezing [20, 24, 25]. In this study, empirical-ethical research was used to identify the viewpoints of women in Austria on egg freezing by asking them to reflect in depth upon the issues involved. Our study adds to the debate by providing the lay persons’ perspectives, for example regarding interlinked normative concepts of the life-course, biomedicalization and reproductive autonomy. This will be discussed further in the following, after giving a short summary of the viewpoints identified.

In this study we identified three prevailing viewpoints on egg freezing among women in Austria. The first one *(“Women should decide for themselves”*) can be described as a viewpoint with a strong emphasis on individual choices without outside interference. The right to have a child is valued highly in this regard, yet, women with this viewpoint seem indifferent toward regulatory intervention (by policy or employer) to facilitate childbearing at younger age. Viewpoint one therefore perceives egg freezing as an option to claim the right to a child. The second viewpoint (*“We should accept nature but change policy”*) clearly expresses a critical opinion on assisting reproduction with technology, particularly when non-medical reasons are involved. It stresses the need for changes in policy in order to facilitate a good work-life balance. Viewpoint two therefore sees policy intervention as the only legitimate way to address and improve the conditions for childbearing and upbringing. The third and final viewpoint identified (*“We need an informed societal debate”*) shows a strongly approving stance towards the use and potential benefits of both medical and non-medical egg freezing. The viewpoint, however, expresses the need for a societal debate and disapproves of the “right” to have a child. Viewpoint three hence considers egg freezing as a responsible choice in the context of socio-cultural changes and does not favour regulatory intervention in this regard.

Our study shows how the use of assisted reproductive technologies is confronted with traditional perceptions of the life course, yet at the same time also challenges them. In this regard, a re-emerging theme in all factors is the experienced challenge of achieving a work-life balance. This, however, must be put in context. Austria can be considered a country where traditional perspectives on the life course and particularly regarding family structures prevail to exist. This is, for example, also reflected in eligibility criteria for assisted reproductive medicine [46], which exclude single women from accessing infertility care. Another example concerns parental leave practice. In Austria, mother and father are entitled to parental leave until the child reaches the age of 24 months. In practice, men generally show very low usage rates of parental leave, while women have longer leave periods; consequently, women with children in Austria more often have part-time work as compared to other European countries [52]. In December 2018, only 3.8% of those receiving parental leave benefits were men (even decreasing from 4.2% in December 2016) [53, 54]. Low usage rates by men, which are due to a complexity of factors that go beyond the scope of this paper, only exemplify the problem of simultaneously managing family and career life for women in Austria. This also needs to be put in context, as norms regarding traditional gender roles and family life seem to be stronger and more prevalent as in other countries [55].

Despite the difficulty of combining family and career being acknowledged in all viewpoints, opinions regarding approaches to address these difficulties were perceived differently across the factors. Viewpoint one emphasized autonomous choices about fertility and seemed fairly indifferent about policy intervention, viewpoint two was in favour of restrictions to reproductive technologies and showed a clear preference for better policies in the labour market as a solution to promote better work-life balance, while viewpoint three saw egg freezing as a real option for women already, it did not favour regulatory intervention, and called for societal debate. Different perceptions on whether, how and by whom measures should be taken also let us reflect upon concepts of autonomy and paternalism. Here, we identify opposing views and a tension between who is supposed to make or be allowed to interfere with reproductive decisions. Literature suggests prioritizing autonomous choices over paternalistic attitudes e.g. from the treating physician [5], but it must be acknowledged that viewpoints favouring paternalistic approaches prevail to exist.

Further, our study illustrates different perceptions as to in how egg freezing interlinks with gender equality. The viewpoints show that egg freezing can be perceived to function as a tool to deal with inequalities women still face in a country like Austria, which ranks lower than the European average on the gender equality index (AT = 65.3, EU28 = 67.4) [55]. In viewpoint three, engaging in egg freezing is therefore perceived as making responsible choices in context of socio-cultural changes and the pressures resulting from them, which relates to what Carroll and Kroløkke [23] frame as “enacting responsible reproductive citizenship”. Yet, despite the potential positive effects egg freezing might create in terms of reproductive justice [19], considering it as an enabler of greater equality in society can also be problematic [10]. While the potential benefits of egg freezing should not be withhold by restricting access to it, focusing on the root causes of postponed childbearing and making necessary changes for working mothers is still inevitable [5].

Finally, we want to add with our study that not only egg freezing itself, but also the diversity of views on it creates tensions. Women who consider or engage in egg freezing are hence not only confronted with the ethical issues already identified in the literature, yet in practice they are also confronted with further issues related to conflicting normative concepts they are faced with in daily-life. These include potential pressure to engage in egg freezing as a “responsible citizen”, unfulfilled expectations in terms of success rates, and/or through stigma women experience when being confronted with stereotypical portrayals [56, 57] their environment may hold. Hence, adding to the psychological benefit of egg freezing in terms of relief from pressure from the biological clock [14], egg freezing might also create psychological harm in various other ways, which needs to be considered more in the general discourse on egg freezing. We must add at this point that our study, however, reflects in general women’s viewpoints. Viewpoints of men or other groups may differ from these and might create even further (or other) tensions in that regard.

Our study also has limitations which need to be reflected upon. First, developing a comprehensive statement set was not without challenges. Covering the whole spectrum of the discussion on egg freezing was crucial for the success of this study. We are aware that the statements are formulated and can be interpreted differently, also in relation to the other statements, which made the provision of comments by the respondents even more important to put their rankings of the statements in perspective. Second, we conducted this study online. On the one hand, this allowed us to conduct the study remotely and to reach out to a larger and diverse sample more easily. On the other hand, this also had several limitations. It was not possible for respondents to ask questions while completing the ranking exercise, which may have led to drop-outs. However, this could not be monitored with the software we used. It also did not allow us to ask in-depth follow-up questions. Although we collected written explanations of the rankings from respondents, an in-person interview might have resulted in even richer qualitative data to help interpret the factors. In addition, due to the approach we took to recruitment of respondents and data collection, it is likely that only women with adequate IT infrastructure and the motivation to engage in this online set-up took part in the study, which could mean that women with a strong interest in the topic are overrepresented. Third, as Table 1 shows, most participating women were between 26 and 40 years old and had a high educational level. In the end, the respondents we have recruited for this study may represent the most relevant population for investigating this topic, as it is likely that the topic has most actual relevance for this group and, correspondingly, there was more interest to participate in the study. The demographic profile of the sample also shows that we did not account for transgender participants. However, trans or non-binary people who may not identify as either male or female, or who might identify as male but have had their eggs frozen are for this reason not included in this study. This must be considered a limitation of our study. Future research should therefore take this into consideration. Finally, we are aware that exclusively asking women about their opinion does not reflect a holistic societal perspective. However, as women are the ones who are targeted and immediately affected by the technology, we considered it relevant to investigate their viewpoints first. Future research on the same or similar topics should consider including other stakeholders’ perspectives as well, like men, representatives from special interest groups, employers or policy makers. This may provide even richer insights, but also reveal what the consensus is on this topic between all these stakeholders, and what distinguishes their views most importantly.

## Conclusion

Conducting this study allowed us to explore women’s viewpoints on egg freezing in a country where this technology is currently not allowed for non-medical or social reasons. Our research adds to the discussion on the ethics of assisted reproduction—and egg freezing in particular—by providing empirical insights into how the technology itself, but also its implications, are perceived by women in Austria. As we showed in this paper, three distinct viewpoints exist with different prioritizations of values. It also illustrates the normativity surrounding egg freezing, upon which we reflected hereabove. This study therefore adds to the larger debate on assisted reproductive technologies and egg freezing, as it provides in-depth insights into the realities of those directly affected by the technology: women. Following up on the lay persons perspective is particularly important in the context of future innovations in health care and other fields, which will continue to challenge established standards and create new normative tensions. As we see, regulations do not necessarily match with the lived realities of those affected by the rules. Decision makers therefore need to (re)consider whether restricting access to health care innovations that are nonetheless available across the border is a safe, cost-effective, and finally ethically sound policy.

## Data Availability

The datasets used and/or analysed during the current study are available from the corresponding author on reasonable request.

## Abbreviations

ART: Assisted reproductive technology
ASRM: American Society for Reproductive Medicine
EIGE: European Institute for Gender Equality
ESHRE: European Society of Human Reproduction and Embryology
FMedG: Fortpflanzungsmedizingesetz (‘Law on Reproductive Medicine’)
FMedRÄG: Fortpflanzungsmedizinrechts-Änderungsgesetz (‘Amendment to the Law on Reproductive Medicine’)
IVF: In vitro fertilization
Resp: Respondent

## Footnotes

1 Over the years, “Social Egg Freezing” has established as a term used in Austrian media coverage (e.g. DER STANDARD *“Freezing eggs: postponing motherhood”* 8 Dec. 2019; NEWS *“Social Egg Freezing—Pro or Con?”* 27 Oct. 2014; DIE PRESSE “Social Egg Freezing—kids from the cold” 4 Aug. 2012 etc.).

2 “Meryns Sprechzimmer: Kinderwunsch auf Eis—Baby auf Bestellung?” broadcasted on the Austrian public television station ORF on 11 June 2019.

3 See www.hackert.biz/flashq

4 Please note that we used a sorting grid ranging from 1 to 9 during the data collection in order to emphasize only the order of the columns, whereas we used the scaling of -4 to + 4 in the analysis to better illustrate the opposing sides (with 0 as neutral).

## References

[1] Mills M, Rindfuss RR, McDonald P, te Velde E, on behalf of the ESHRE Reproduction and Society Task Force. Why do people postpone parenthood? Reasons and social policy incentives. Hum Reprod Update. 2011;17(6):848–60. 10.1093/humupd/dmr026 PMC3529638 21652599

[2] Bozzaro C (2018). Is egg freezing a good response to socioeconomic and cultural factors that lead women to postpone motherhood?. Reprod Biomed Online.

[3] Eurostat. Mean age of women at childbirth and at birth of first child. https://ec.europa.eu/eurostat/tgm/refreshTableAction.do?tab=table&plugin=1&pcode=tps00017&language=en. 2018.

[4] ASRM, SART. Mature oocyte cryopreservation: a guideline. Fertil Steril. 2013;99(1):37–43. 10.1016/j.fertnstert.2012.09.028 23083924

[5] ESHRE Task Force on Ethics and Law, including, Dondorp W, de Wert G, Pennings G, Shenfield F, Devroey P, et al. Oocyte cryopreservation for age-related fertility loss. Hum Reprod. 2012;27(5):1231–7. 10.1093/humrep/des029 22357771

[6] Business Insider. What you need to know about egg-freezing, the hot new perk at Google, Apple, and Facebook. 2017 Sep 19 [cited 2020 Sept. 19]; Available from: https://www.businessinsider.com/egg-freezing-at-facebook-apple-google-hot-new-perk-2017-9?r=DE&IR=T.

[7] Daniluk JC, Koert E (2016). Childless women’s beliefs and knowledge about oocyte freezing for social and medical reasons. Hum Reprod.

[8] Wennberg A-L, Rodriguez-Wallberg KA, Milsom I, Brännström M (2016). Attitudes towards new assisted reproductive technologies in Sweden: a survey in women 30–39 years of age. Acta Obstet Gynecol Scand.

[9] Singer P, Wells D (1983). In vitro fertilisation: the major issues. J Med Ethics.

[10] Harwood K (2009). Egg Freezing: a breakthrough for reproductive autonomy?. Bioethics.

[11] Weber-Guskar E (2018). Debating social egg freezing: arguments from phases of life. Med Health Care Philos.

[12] Klitzman R (2016). Buying and selling human eggs: infertility providers’ ethical and other concerns regarding egg donor agencies. BMC Med Ethics.

[13] Vanstone M, Cernat A, Nisker J, Schwartz L (2018). Women’s perspectives on the ethical implications of non-invasive prenatal testing: a qualitative analysis to inform health policy decisions. BMC Med Ethics.

[14] Mertes H. Egg banking in anticipation of age-related fertility decline : using medical technology for better, not for worse. Ghent; 2017.

[15] Wiesing U (2019). Egg freezing: a new medical technology and the challenges of modernity. Bioethics.

[16] Gruben V. Freezing as Freedom? A Regulatory Approach to Elective Egg Freezing and Women’s Reproductive Autonomy. Alta Law Rev [Internet]. 2017 Mar 31 [cited 2020 Sep 19]; Available from: http://albertalawreview.com/index.php/ALR/article/view/773.

[17] Dondorp WJ, De Wert GMWR (2009). Fertility preservation for healthy women: ethical aspects. Hum Reprod.

[18] Mertes H, Pennings G (2011). Social egg freezing: for better, not for worse. Reprod Biomed Online.

[19] Goold I, Savulescu J (2009). In favour of freezing eggs for non-medical reasons. Bioethics.

[20] Baldwin K (2018). Conceptualising women’s motivations for social egg freezing and experience of reproductive delay. Sociol Health Illn.

[21] Inhorn MC, Birenbaum-Carmeli D, Birger J, Westphal LM, Doyle J, Gleicher N (2018). Elective egg freezing and its underlying socio-demography: a binational analysis with global implications. Reprod Biol Endocrinol.

[22] Kılıç A, Göçmen İ (2018). Fate, morals and rational calculations: freezing eggs for non-medical reasons in Turkey. Soc Sci Med.

[23] Carroll K, Kroløkke C (2018). Freezing for love: enacting ‘responsible’ reproductive citizenship through egg freezing. Cult Health Sex.

[24] Gürtin ZB, Morgan L, O’Rourke D, Wang J, Ahuja K (2019). For whom the egg thaws: insights from an analysis of 10 years of frozen egg thaw data from two UK clinics, 2008–2017. J Assist Reprod Genet.

[25] Baldwin K, Culley L, Hudson N, Mitchell H, Lavery S (2015). Oocyte cryopreservation for social reasons: demographic profile and disposal intentions of UK users. Reprod Biomed Online.

[26] Stoop D, Nekkebroeck J, Devroey P (2011). A survey on the intentions and attitudes towards oocyte cryopreservation for non-medical reasons among women of reproductive age. Hum Reprod.

[27] Hodes-Wertz B, Druckenmiller S, Smith M, Noyes N (2013). What do reproductive-age women who undergo oocyte cryopreservation think about the process as a means to preserve fertility?. Fertil Steril.

[28] de Groot M, Dancet E, Repping S, Goddijn M, Stoop D, van der Veen F (2016). Perceptions of oocyte banking from women intending to circumvent age-related fertility decline. Acta Obstet Gynecol Scand.

[29] O’Brien Y, Martyn F, Glover LE, Wingfield MB (2017). What women want? A scoping survey on women’s knowledge, attitudes and behaviours towards ovarian reserve testing and egg freezing. Eur J Obstet Gynecol Reprod Biol.

[30] Tozzo P, Fassina A, Nespeca P, Spigarolo G, Caenazzo L (2019). Understanding social oocyte freezing in Italy: a scoping survey on university female students’ awareness and attitudes. Life Sci Soc Policy.

[31] Schick M, Sexty R, Ditzen B, Wischmann T (2017). Attitudes towards Social Oocyte Freezing from a Socio-cultural Perspective. Geburtshilfe Frauenheilkd.

[32] Salloch S, Schildmann J, Vollmann J (2012). Empirical research in medical ethics: How conceptual accounts on normative-empirical collaboration may improve research practice. BMC Med Ethics.

[33] Borry P, Schotsmans P, Dierickx K (2005). The birth of the empirical turn in bioethics. Bioethics.

[34] Wangmo T, Hauri S, Gennet E, Anane-Sarpong E, Provoost V, Elger BS (2018). An update on the “empirical turn” in bioethics: analysis of empirical research in nine bioethics journals. BMC Med Ethics.

[35] Calhaz-Jorge C, De Geyter Ch, Kupka MS, Wyns C, Mocanu E, Motrenko T, Scaravelli G, Smeenk J, Vidakovic S, Goossens V. Survey on ART and IUI: legislation, regulation, funding and registries in European countries. Hum Reprod Open. 2020 Feb;1–15. 10.1093/hropen/hoz044 PMC7002185 32042927

[36] Hammami MM, Abuhdeeb K, Hammami MB, De Padua SJS, Al-Balkhi A (2019). Prediction of life-story narrative for end-of-life surrogate’s decision-making is inadequate: a Q-methodology study. BMC Med Ethics.

[37] McHugh N, Baker RM, Mason H, Williamson L, van Exel J, Deogaonkar R (2015). Extending life for people with a terminal illness: a moral right and an expensive death? Exploring societal perspectives. BMC Med Ethics.

[38] Truijens D, van Exel J. Views on deceased organ donation in the Netherlands: A q-methodology study. Rabinowitz M, editor. PLOS ONE. 2019 May 24;14(5):e0216479. 10.1371/journal.pone.0216479 PMC6534345 31125339

[39] FmedG. BGBl. 1992.

[40] IVF Fonds Act. BGBl. 1999.

[41] Flatscher-Thöni M, Voithofer C (2017). Eizellenspende und PID: Offene Fragen des FMedRÄG 2015. Imago Hominis.

[42] OEGGG Österreichische Gesellschaft für Gynäkologie und Geburtshilfe. Stellungnahme zur Novellierung des Fortpflanzungsmedizingesetzes (FMedG) und des IVF-Fondes-Gesetzes. OEGGG; 2014.

[43] Kopetzki C (2014). Social Egg Freezing. Recht Med.

[44] Kostenzer J (2020). Eizellen einfrieren für später? Die Kontroverse um Social Egg Freezing in Österreich. Z Für Krit Recht Ges.

[45] Austrian Bioethics Commission. Stellungnahme der Bioethikkommission beim Bundeskanzleramt zum Entwurf eines Bundesgesetzes, mit dem das Fortpflanzungsmedizingesetz, das Allgemeine bürgerliche Gesetzbuch und das Gentechnikgesetz geändert werden (Fortpflanzungsmedizinrechts-Änderungsgesetz 2015—FMedRÄG 2015). Bioethikkommission beim Bundeskanzleramt; 2014.

[46] FMedRÄG. BGBl. 2015.

[47] Culley L, Hudson N, Rapport F, Blyth E, Norton W, Pacey AA (2011). Crossing borders for fertility treatment: motivations, destinations and outcomes of UK fertility travellers. Hum Reprod.

[48] Watts S, Stenner P (2012). Doing Q methodological research: theory, method and interpretation.

[49] Pennings G (2013). Ethical aspects of social freezing. Gynécologie Obstétrique Fertil.

[50] Kostenzer J, Bos AME, de Bont A, van Exel J (2021). Unveiling the controversy on egg freezing in the Netherlands: A Q-methodology study on women’s viewpoints. Reprod Biomed Soc Online..

[51] Schmolck P, Atkinson J. PQ method software and manual, PQMethod 2.35 with PQROT 2.0 (10-Nov-2014) [Internet]. 2018 [cited 2019 Dec 10]. Available from: http://schmolck.org/qmethod/.

[52] Bundesministerium für Arbeit, Soziales, Gesundheit und Konsumentenschutz. Elternkarenz [Internet]. 2019 [cited 2020 Jan 13]. Available from: https://www.sozialministerium.at/Themen/Arbeit/Arbeitsrecht/Karenz-und-Teilzeit/Elternkarenz.html.

[53] Statistik Austria. Vereinbarkeit von Beruf und Familie [Internet]. 2019 [cited 2020 Jan 27]. Available from: ttp://www.statistik.at/web_de/statistiken/menschen_und_gesellschaft/soziales/gender-statistik/vereinbarkeit_von_beruf_und_familie/index.html.

[54] DER STANDARD. Zahl der Väter in Karenz geht zurück. 2018 [cited 2020 Jan 14]; Available from: https://www.derstandard.at/story/2000087803288/weniger-maenner-in-vaeterkarenz.

[55] EIGE. Gender equality index 2019: work–life balance. [Internet]. LU: EIGE; 2020 [cited 2020 Sep 19]. Available from: https://data.europa.eu/doi/10.2839/319154

[56] Martin LJ (2010). Anticipating infertility: egg freezing, genetic preservation, and risk. Gend Soc.

[57] Mertes H (2013). The portrayal of healthy women requesting oocyte cryo-preservation. Facts Views Vis ObGyn.

